# Medical Augmentation (Med-Aug) for Optimal Data Augmentation in Medical Deep Learning Networks

**DOI:** 10.3390/s21217018

**Published:** 2021-10-22

**Authors:** Justin Lo, Jillian Cardinell, Alejo Costanzo, Dafna Sussman

**Affiliations:** 1 Electrical, Computer and Biomedical Engineering, Ryerson University, Toronto, ON M5B 2K3, Canada; justin1.lo@ryerson.ca (J.L.); jillian.cardinell@ryerson.ca (J.C.); acostanzo@ryerson.ca (A.C.); 2 Institute for Biomedical Engineering, Science and Technology (iBEST) at Ryerson University & St. Michael’s Hospital, Toronto, ON M5B 1T8, Canada; 3 The Keenan Research Centre for Biomedical Science, St. Michael’s Hospital, Toronto, ON M5B 1T8, Canada; 4 Department of Obstetrics and Gynecology, Faculty of Medicine, University of Toronto, Toronto, ON M5G 1E2, Canada

**Keywords:** deep learning, data augmentation, segmentation, fetal MRI, convolutional neural networks

## Abstract

Deep learning (DL) algorithms have become an increasingly popular choice for image classification and segmentation tasks; however, their range of applications can be limited. Their limitation stems from them requiring ample data to achieve high performance and adequate generalizability. In the case of clinical imaging data, images are not always available in large quantities. This issue can be alleviated by using data augmentation (DA) techniques. The choice of DA is important because poor selection can possibly hinder the performance of a DL algorithm. We propose a DA policy search algorithm that offers an extended set of transformations that accommodate the variations in biomedical imaging datasets. The algorithm makes use of the efficient and high-dimensional optimizer Bi-Population Covariance Matrix Adaptation Evolution Strategy (BIPOP-CMA-ES) and returns an optimal DA policy based on any input imaging dataset and a DL algorithm. Our proposed algorithm, Medical Augmentation (Med-Aug), can be implemented by other researchers in related medical DL applications to improve their model’s performance. Furthermore, we present our found optimal DA policies for a variety of medical datasets and popular segmentation networks for other researchers to use in related tasks.

## 1. Introduction

DL has become a popular subdivision of artificial intelligence and has shown great success in many medical image analysis tasks [1,2,3]. DL algorithms have become more cost effective and accurate, making them an ideal candidate for improving clinical workflows [3]. However, there are still many limitations to DL networks that restrict their potential range of applications. One of the primary issues, especially in clinical applications, is their reliance on large datasets. DL networks need diverse and extensive datasets to develop robust and flexible networks [1]. Particularly in medical studies, datasets are limited due to privacy and legal concerns, disease frequency, cost of data acquisition, and the time-consuming labelling process [1,2,3]. Despite these constraints, the success of DL networks in medicine has motivated work to adapt these networks to function well under low-data circumstances [1]. Data augmentation (DA) has been a common method used to combat this data limitation restriction.

A popular method of DA is randomized DA, but studies have shown that randomized DA does not accurately synthesize the random occurrences and true variability that help improve the robustness of the networks. These types of random policies can also be highly sensitive to the parameters chosen [4]. Random augmentations can potentially compile transformations that are too intense or too subtle to improve network function [5]. Certain augmentations can drastically decrease DL model performance. A clear example would be with the MNIST dataset of hand-written numbers, in which random flips or rotations could completely change the number or even make them into a non-existent symbol [6]. Random augmentations are often picked by trial and error or learned through the experience of the researcher, and with this method, there is no way to know whether the augmentations improved training until after it is complete [6]. This random selection can lead to redundant augmentations that can potentially introduce biases into the network or slow training [7]. Previous studies have found that using learned DA policies noticeably improves performance of networks relative to random DA policies [4,8]. Another study with a VGG network found that DA influenced both discriminative and generative learning [9]. Many of these studies emphasize the importance in careful selection of DA policies, creating a need for viable tactics in optimizing and learning DA policies.

One of the earliest models in learning DA policies was AutoAugment. AutoAugment models the final accuracy of the target neural network as a function of the DA policies applied to the training set. AutoAugment uses a recurrent neural network (RNN) and a Proximal Policy Optimization (PPO) algorithm to optimize this function. AutoAugment was shown to improve performance of classification networks with the learned policies; however, the search process required 15,000 iterations and required prolonged time and computational power [10]. BO-Aug used the same basic structure as AutoAugment but used Bayesian Optimization (BO) to optimize the function. BO-Aug could attain results with fewer iterations and less computational load [11]. Other modifications to the AutoAugment approach have been proposed to expand applications to bounding box problems or improve training time [7,8]. Population-Based Augmentation (PBA) attempted a DA policy schedule such that a new policy was selected for each epoch. This method, although faster, could not out-perform AutoAugment [7].

Previous methods are limited to the computational power that is needed. Obtaining DA policies could take several weeks even on high end machines. In our study, we present a DA policy search algorithm that is computationally efficient compared to other state-of-the-art models and is tailored towards medical image segmentation. We adopt the basic design proposed in AutoAugment and BO-Aug by treating the accuracy of the neural network as a function of the DA policies applied to the training set. Many of the algorithms use only basic transformations which cannot mimic the unique variations within medical images [2]. To combat this issue, we consider an extended set of transformations, including MRI k-space based transformations, to accommodate the variations that occur in real medical MRI datasets. We implement the efficient high-dimensional optimizer Bi-Population Covariance Matrix Adaptation Evolution Strategy (BIPOP-CMA-ES) to search through the extended set of transformations. We refer to our algorithm as Medical Augmentation (Med-Aug). With this strategy and transformation set, we can return optimal DA policies based on commonly used medical datasets and base architectures, allowing for generalizability and reusability of resulting policies on similar problems.

The function of the network accuracy performance with respect to the DA policy is the objective function of our problem and is optimized using BIPOP-CMA-ES. BIPOP-CMA-ES is an advanced version of CMA-ES that incorporates an alternative restart strategy. The basic CMA-ES algorithm is a continuous, stochastic, population-based search method commonly applied to black box functions. Samples in the search space are selected using multivariate normal distributions and covariance matrices. The best solutions in this sample space are used to update the distribution pattern criteria for the selection of the next sample space to converge on the optimum. This base algorithm has only one population size for every sample and is most useful for local search [12]. BIPOP-CMA-ES modifies this strategy by incorporating restarts and multiple population sizes.

In this work, we present an algorithm, Med-Aug, that can be used to combat the data limitations in medical DL studies by finding the DA policies that lead to the ideal performance of a target network. We ran our Med-Aug algorithm in a series of experiments using some of the most popular medical segmentation models as our target networks with the Brain Tumor Segmentation (BraTS) dataset [13,14,15], Medical Decathlon Cardiac Dataset [16], and our own anonymized fetal MRI dataset. Both BraTS and the Medical Decathlon Cardiac Dataset are open-access datasets used for medical imaging research, which did not require ethics approval. Ethics approval for the anonymized fetal MRI dataset was provided by our institution. This work presents technical improvements to the previous state of the art, BO-Aug, by adding advanced medical-specific transformations and improving the search strategy. Furthermore, we provide the optimal DA policies found from our experiments for other researchers in related clinical DL applications to improve their segmentation networks.

## 2. Materials and Methods

In this algorithm, we search for a combination of image augmentations applied to a training set that results in the highest test performance of the neural network. Our implementation follows a similar policy and augmentation format to BO-Aug and AutoAugment [10,11]. An image augmentation policy is composed of three sub-policies, each of which consists of two transforms, and the transforms are each associated with a probability and magnitude.

**Figure 1:**
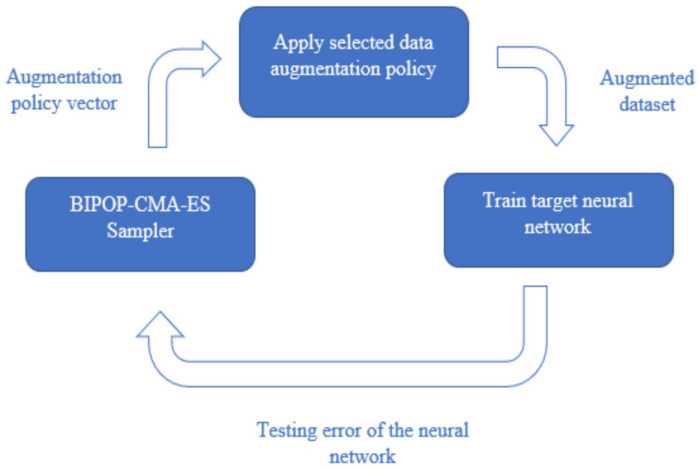
Workflow of the data augmentation optimization process.

To perform the search, we optimize a black box function with the augmentation policy as the input, and the output being the error of the neural network. In this study, we minimize the black box function using BIPOP-CMA-ES to find the optimal DA policy [17,18]. This process is illustrated in Figure 1.

### 2.1. Augmentation Policies

The set of image augmentations included in the search space consists of transformations from PIL, imgaug, and TorchIO [2,19,20]. The transforms were selected to mimic real variability in medical datasets produced by anatomical differences or artefacts, as well as to improve robustness to image property variability such as brightness and contrast values. We selected general transformations to accommodate this variability while avoiding transformations that may cause issues in certain medical datasets, such as horizontal and vertical flips which can be problematic in tasks where relative position is an important marker.

**Figure 2:**
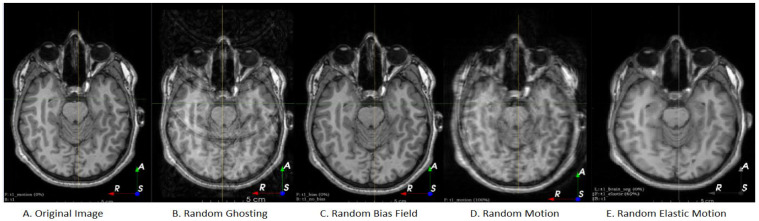
Extended set of transformations specifically used for biomedical imaging applications.

The transformations are each associated with a magnitude and probability. Each transformation has an execution probability between 0 and 1, and the magnitude parameter is set between 0 and 9 [11]. Within each transform function, the magnitude parameter is normalized such that the minimum magnitude still creates a visually noticeable change, and the maximum magnitude is set to prevent distorting the image beyond recognition. Certain transformations cannot utilize a magnitude parameter, including AutoContrast, Equalize, and Invert. The full list of transforms is presented in Table 1. There are 17 transformations to select from, and two transformations applied consecutively make up a sub-policy, resulting in a total of 289 sub-policies to search through. Figure 2 illustrates the extended set of MRI-based DA added to the set. Our search algorithm selects three sub-policies to form a full augmentation policy. When the augmentation policy is applied, each image is augmented with each sub-policy, creating three new images in addition to the original. The process of applying a DA policy to an image is outlined in the below Algorithm 1. When applying each sub-policy, a transformation on the input image is only applied if the probability is less than the normal random variable. If the random number is greater than the probability, the algorithm simply proceeds to the following transformation in the subpolicy. The random variables are re-sampled at every iteration of the for-loop for every new image. This algorithm is repeated a total of three times for each sub-policy.
**Algorithm 1:** Process for applying a data augmentation policy
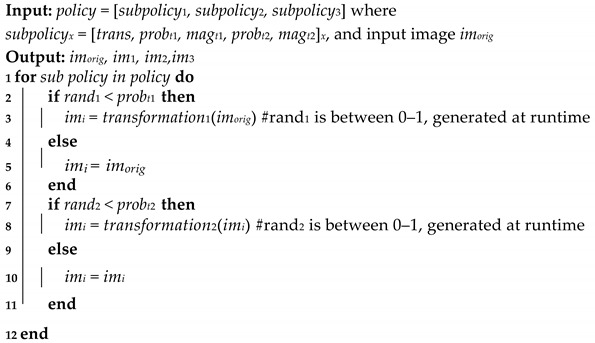


The described optimization system was run using the popular clinical dataset Brain Tumour Segmentation (BraTS) as the input data. The BraTS dataset is a multimodal volumetric dataset containing labelled T1, T1ce, T2, and FLAIR images for each patient. The dataset consists of 285 patients. The data are labels including edema, non-enhancing solid core, necrotic/cystic core, and enhanced core [13,14,15]. Due to computational concerns and processing limitations, we used only one modality (MRI sequence), sliced along the z-axis to obtain 2D images for segmentation. We opted to use the FLAIR modality as it shows the whole tumor region for segmentation [13]. However, while searching for the DA policy, we only used 60% of this dataset to simulate a low data environment. The cardiac dataset from The Medical Segmentation Decathlon consisted of 30 3D volumes. MRI scans of the entire heart were acquired during a single cardiac phase. The region of interest (ROI) was the left atrium [16].

In addition to the BraTS and cardiac dataset we also used our own fetal dataset. De-identified patient data of whole-body fetal MRIs were acquired using various sequences as part of several previous studies (The Hospital for Sick Children, Toronto, Canada). An SSFP sequencer was used on a 1.5T scanner and a 3D SSFP with SENSE along 2D (CHOP sequence [21]) was used on a 3.0T scanner. Healthy developing fetuses were used. Average gestational age was 28.42 ± 4.62 weeks. Segmentation was conducted by a collaborating radiologist.

### 2.2. Experiments

The proposed method was trained and tested on an Nvidia RTX 2070 Super graphics card with an 8 GB GDDR6 video memory, 2560 stream processors, 1605 MHz base clock, and a 1770 MHz boost clock. The CPU used was an Intel Core i7-9700 CPU at 3.00 Ghz clock speed with 16 GB of DDR4 ram. Four widely-used 2D segmentation models were studied as our target networks: U-Net, Residual U-Net with four residual blocks, V-Net, and SegResNet. These networks were implemented using the PyTorch-based API, MONAI [22]. The search algorithm was run with each of the described networks, resulting in four different corresponding DA policies per dataset. The performance of the models trained with the resulting optimal DA policies from Med-Aug was then compared to the models trained with other DA methods.

Three datasets were experimented with: (1) BraTS, (2) Cardiac MRI, and (3) Fetal MRI to evaluate the performance of our DA search algorithm. We also compared our proposed approach with BO-Aug. BO-Aug is another DA search algorithm that searches through a set of more general computer vision transformations and employs Bayesian optimization as the black box optimizer. The segmentation performance for all our experiments was measured using the Dice Similarity Coefficient (DSC). The hyperparameters for each neural network remained consistent. This may have lowered the performance of a particular algorithm relative to performance in the literature; however, these parameters were kept consistent so that changes in performance could be attributed to the change in DA policy, rather than to the potential impact of changing hyperparameters.

### 2.3. Model Parameters

All neural networks were implemented using MONAI’s built-in networks, without modification [23]. Each network was initialized randomly and trained using losses from MONAI. For both the plain U-Net and the Residual U-Net experiments, the networks were trained for 50 epochs during the DA search with a learning rate of 1 × 10^4^. SegResNet was trained for 50 epochs using a learning rate of 1 × 10^5^ as preliminary experiments and readings indicated this network required a lower learning rate. During preliminary experiments, we noted that the V-Net algorithm generally took more epochs to achieve testing performance on par with the other deep learning networks used in the experiments; therefore, we used 100 epochs and a learning rate of 1 × 10^4^ during the DA search. All networks were trained using Tversky Loss and the Adam optimizer. Once the DA search was complete, the networks were re-trained from scratch using the suggested optimal policies. They were trained with the same hyperparameters, except for the number of epochs. Epochs were reduced during the search to limit the search time. While training networks on the optimal DA policies, 100 epochs were used for U-Net, Residual U-Net, and SegResNet. 150 epochs were used for V-Net. The losses, learning rate, and optimizers were all kept constant.

To find the optimal DA policy for each neural network, we ran the BIPOP-CMA-ES search with a maximum of 200 function evaluations, 9 restarts, and a function target error of 0.2. Table 2 shows the performance of four architectures with (1) no DA, (2) basic set of DA, (3) the optimal policies found from our Med-Aug search algorithm, and (4) the optimal policies found from BO-Aug. Table 2 demonstrates that any form of DA resulted in an improvement in performance. The 2nd column in Table 2 shows that the random basic DA often only lead to improvements in the 1–3% range, whereas columns 3 and 4 showed the strategically selected policies lead to improvements of up to 10%. The performance with the Med-Aug policies was competitive if not better than with the BO-Aug policies. SegResNet showed the most drastic increase in performance with using Med-Aug compared to BO-Aug, and the fetal dataset consistently showed higher performance with Med-Aug. A 95% confidence interval and a significance level of *p* < 0.05 was used for our statistical analysis. If the *p*-values were found to be less than 0.05, this demonstrates that the difference in DSC performance between using a basic set of DA and optimal policies are significant.

## 3. Results

**Figure 3:**
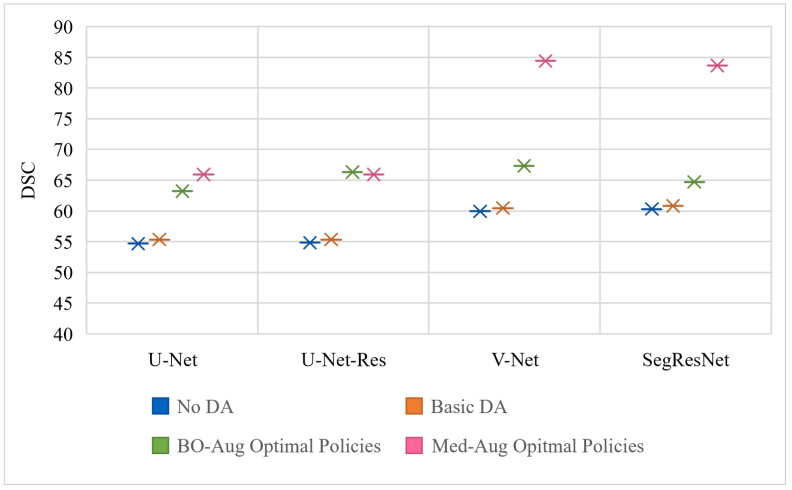
Visualization of the testing performance in DSC of four different neural networks using different DA policies on the training set for the BraTS data experiments.

**Figure 4:**
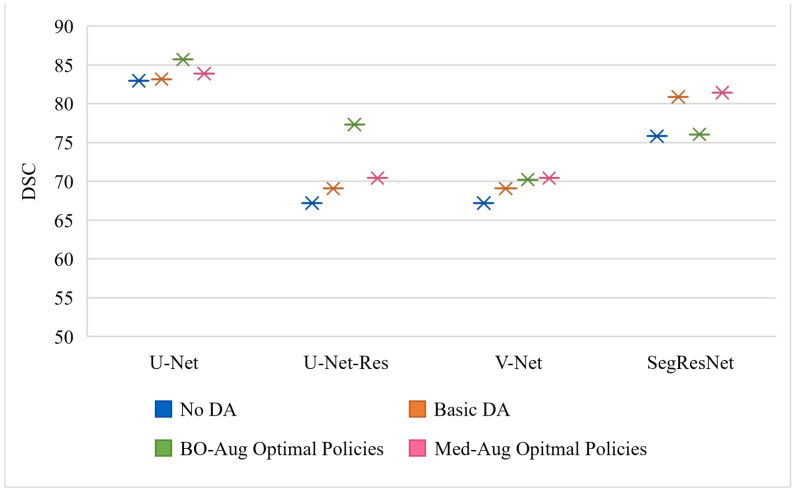
Visualization of the testing performance in DSC of four different neural networks using different DA policies on the training set for the Cardiac data experiments.

**Figure 5:**
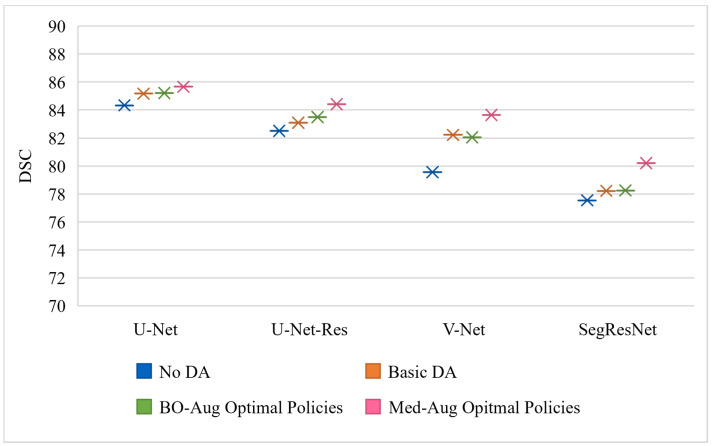
Visualization of the testing performance in DSC of four different neural networks using different DA policies on the training set for the Fetal data experiments.

Table 2 shows the performance of the neural networks on the varying datasets with different DA methods used on the training set. Figure 3, Figure 4 and Figure 5 graphically show the data from Table 2. Figure 3 is trained and evaluated on the BraTS dataset, Figure 4 is trained and evaluated on the Cardiac dataset, and Figure 5 is trained and evaluated on the Fetal MRI dataset.

Illustrated in Table 3 are the optimal policies found using our medical DA search with the BraTS dataset for the four models. For example, the optimal policies found corresponding to the U-Net model with BraTS was as follows: Sharpness and Rotation, Invert and Shear, and Elastic Deform and Brightness. Transform 1 and Transform 2 are applied consecutively and based on the probability. The process of applying a sub-policy was as follows: two random variables were generated separately between 0 and 1. If the value of the first random variable was less than the probability of the first transformation in the sub-policy, the transformation was applied. This process was then repeated for Transform 2, and the transform is applied to the resulting image from Transform 1. Therefore, for any single sub-policy, up to two transformations can be applied consecutively for one image at varying magnitudes. The optimal policies found by our Med-Aug search for the other datasets can be found in the appendix (Appendix B and Appendix C).

Table 4 shows the statistical significance of the performance difference between basic DA and Med-Aug and BO-Aug. An independent *t*-test was calculated on the DSC values from the different testing performances shown in Table 1.

Table 5 shows the transferability of the optimal policies learned from Med-Aug to other networks. We evaluated transferability of the optimal policies learned from the BraTS experiments. The optimal policies learned from the BraTS and the U-Net search were applied to the other neural networks during their training on BraTS and the final testing performance was recorded. This process was repeated for the optimal policies learned from BraTS and all other networks, so the policies learned from the V-Net BraTS search were applied to U-Net, U-Net-Res, SegResNet, and so on. The diagonal of Table 5 represents the performance of the neural networks that used the optimal DA policy that corresponded to their own search, i.e., U-Net trained using the U-Net found policies. This table shows that the networks generally do best with their corresponding optimal policies; however, the performance degradation from using the incorrect optimal policy is minimal indicating that similar tasks and networks can benefit from the policies found in this study.

Table 6 illustrates the computational time required to determine the optimal policy using BraTS and reduced BraTS dataset. As shown, the average computational time on the original BraTS dataset was approximately 274 hours (~11 days). However, the reduced BraTS dataset also obtained comparable segmentation performance while only taking 1/4 of the average computational time. This indicated that our search algorithm was able to perform well under reduced data conditions and still result in increased network performance. This is especially beneficial for those who do not have a large data set to perform the optimal policy search on.

## 4. Discussion

In this paper, we presented a novel approach for optimizing DA for medical imaging datasets using transformations that are suitable to medical studies. We experimented with three datasets: BraTS, a public Cardiac MRI dataset, and a private fetal MRI dataset. The cardiac and fetal datasets were both relatively small datasets, adequately simulating a real-world situation as medical datasets are costly to obtain and are limited by the occurrence of the pathology being studied. For the BraTS dataset, which is relatively large, we simulated a low data environment by using only 60% of the training data. Conducting experiments in a low-data environment allows these experiments to show the impact of optimized DA in a restrained environment where DA is needed the most. We also studied our DA policies across four different popular medical segmentation networks to present diverse and thorough results on DA and medical imaging tasks.

To evaluate the effects of DA, three experiments were conducted: (1) Evaluating the DSC when no DA is applied, (2) evaluating the DSC when a basic set of DA is applied, (3) evaluating the DSC using the optimal policy from Med-Aug, and (4) DSC when using optimal policies from BO-Aug, a non-medical search strategy. The results demonstrated that even the use of the most basic DA had significant impact on the performance of the model, as shown in Table 1; however, strategic policies selected by either BO-Aug or our Med-Aug show consistent improvements over random DA. This finding backs previous studies which have found that strategic DA policies improve performance relative to random DA policies [4,8]. Although both BO-Aug and Med-Aug offer benefits to performance, Med-Aug was consistently competitive or out-performed BO-Aug.

BO-Aug was used as a baseline for these experiments as Med-Aug was designed off its framework but tailored for medical imaging [11]. The main difference between our implementation and BO-Aug was the number of required function evaluations and the set of transformations to select from. With fewer function evaluations and medical-specific transformations, our Med-Aug showed competitive, if not better, performance. In BO-Aug, they ran their search for 100 function evaluations eight separate times, with a total of 800 function evaluations for a given network and dataset combination. They restarted this search manually eight times to offer diversity in their suggested policies; however, 800 evaluations of training neural networks, especially on large 3D medical imaging data, is not feasible for many researchers. By using BIPOP-CMA-ES, restarts are built into the search and are conducted as necessary to converge on the most optimal policy more efficiently. Additionally, rather than rerunning the search on the same data and network combination eight separate times, we ran our search on a variety of medical datasets and networks to develop a deeper understanding of the relation between optimal policies, the data, and the corresponding network.

By testing our search of varied datasets and networks, we were able to observe patterns in the selected optimal policies. We noted that contrast, bias, sharpness, brightness, and elastic deformations are popular transformations across most networks and datasets (see Table 3 and Appendix B and Appendix C). In the cardiac and fetal dataset, we noticed more instances of motion and ghosting artefacts in the optimal policies, compared to BraTS which suggested none. Given that the brain is rigid and both fetal and cardiac imaging are much more prone to motion artefacts, these results are as expected. These patterns stress the importance of knowing one’s own datasets when building a DA policy. The DA policies used should reflect natural variation as well as the technical and biological artefacts that can affect a dataset. This also highlights the issue with using DA policies recommended by pure computer vision research, as in BO-Aug, because the simple transformations cannot capture all the variations that occur in medical images.

BO-Aug and Med-Aug both represent search approaches that optimize neural network performance as a function of the DA used. Other approaches aim towards a single system that jointly optimizes DA with the target network, rather than performing a complete search for policies using the target network accuracy as feedback [5,6]. However, joint-optimization strategies are not easily generalizable because they require joint training of two neural networks (the task network and the search network) for a specific dataset. With our search, we showed that our policies suggested for a given dataset and network combination generalize well across similar networks used with the same dataset, thus eliminating the need to retrain a whole two-part system for any new networks. Alternatively, some researchers developed DL methods to generate new, plausible, and realistic images based on real data and have shown that this method improves performance [4,24,25,26,27]. Although these types of algorithms have shown success in generating new unique samples useful for medical DA, they do not directly consider the effect of augmentation on network performance like our Med-Aug system. Additionally, it can only generate samples similar to those it has already seen, so it may not prepare the network well for a domain shift. Although each type of approach may offer its own benefits, we have shown that our Med-Aug system can recommend versatile and reliable DA policies that lead to consistent improvements in performance for a variety of challenging, small medical datasets.

### Limitations

Our results show that all the DSC scores are <0.7 when using the BraTS dataset, which can be attributed to the fact that each network was not fine-tuned or pretrained. These models were used out-of-box from the MONAI API and were trained with random hyperparameter initialization. To ensure that the performance change from each experiment could be attributed to the change in DA policy, we kept learning rates, optimizers, and epochs the same across the changing DA policies. Changing the training parameters of each model with all the different augmentation policies may have improved performance of each experiment marginally; however, it would be more difficult to identify the impact of changing the DA policy. The absolute DSC performance of these networks should not be taken out of context and should only be considered as comparative numbers to show the impact of DA policies.

## 5. Conclusions

In this paper, we proposed an optimization algorithm that uses BIPOP-CMA-ES optimization to find optimal DA policies for medical DL networks. By using an evolution-based optimization algorithm, we were able to conduct the search with lower computational costs compared to other prominent optimization algorithms. Our algorithm only uses approximately 200 real evaluations at 100 epochs or less per evaluation, which is much fewer than BO-Aug or AutoAugment [10,11]. Additionally, by using medical-specific augmentations and omitting irrelevant computer vision augmentations (such as RGB colour transformations), we showed that our method can result in improved performance in medical DL applications relative to naive random DA.

We present our found optimal data augmentation policies for the four popular algorithms and three demonstrative datasets. These DA policies, or perhaps the most popular or frequently selected transformations, may be used by other researchers with related datasets or networks to improve performance. As the transformations did not change drastically between datasets or architectures, we can recommend that similar policies to any of those found in our paper be implemented in related works.

## Figures and Tables

**Table 1:** Full list of all the transformation operations used in this study.

Operation Name	Description
Shear X/Y	Image shear along the X or Y axis with a rate of *magnitude*
Translate X/Y	Translate image along the X or Y axis by *magnitude* number of pixels
Rotate	Rotate the image by *magnitude* degrees
AutoContrast	Scale the image intensity such that the darkest pixel becomes black, and the lightest pixel becomes white
Invert	Invert the intensities of the image
Equalize	Histogram equalization
Solarize	Invert all pixels above *magnitude* threshold
Posterize	Reduce bits per pixel to *magnitude* bits
Contrast	Adjust contrast by *magnitude*
Brightness	Adjust brightness by *magnitude*
Sharpness	Adjust sharpness by *magnitude*
Random Ghosting	MRI ghosting artefact with *magnitude* number of ghosts
Random Bias Field	MRI magnetic field inhomogeneity with coefficient magnitude of *magnitude*
Random Motion	MRI motion artefact with *magnitude* number of simulated movements
Elastic Transform	Localized movement of pixels using displacement fields with an alpha value of *magnitude*

**Table 2:** Testing performance in DSC of four different neural networks using different DA policies on the training set. (IoU version of the table is found in Appendix A).

Model	Dataset	DSC with no DA (Baseline) (Mean ± Stdv)	DSC with Basic DA (Mean ± Stdv)	DSC with Med-Aug Optimal Policies (Mean ± Stdv)	DSC with BO-Aug Optimal Policies (Mean ± Stdv)
U-Net	BraTS	54.67 ± 0.00011	55.34 ± 0.00093	65.92 ± 0.0026	63.21 ± 0.0088
	Cardiac	82.97 ± 0.017	83.16 ± 0.022	83.89 ± 0.036	85.69 ± 0.029
	Fetal	84.33 ± 0.52	85.17 ± 0.41	85.66 ± 0.55	85.21 ± 0.51
U-Net-Res	BraTS	54.82 ± 0.0011	55.34 ± 0.00093	65.92 ± 0.0034	66.32 ± 0.0069
	Cardiac	67.21 ± 0.091	69.08 ± 0.082	70.42 ± 0.097	77.32 ± 0.019
	Fetal	82.51 ± 0.66	83.09 ± 0.69	84.41 ± 0.77	83.49 ± 0.68
V-Net	BraTS	59.94 ± 0.0026	60.45 ± 0.0016	68.46 ± 0.0036	67.31 ± 0.0076
	Cardiac	67.21 ± 0.028	69.08 ± 0.036	70.42 ± 0.044	70.23 ± 0.061
	Fetal	79.56 ± 0.44	82.22 ± 0.50	83.65 ± 0.51	82.05 ± 0.45
SegResNet	BraTS	60.31 ± 0.0019	60.80 ± 0.0023	68.81 ± 0.0030	64.72 ± 0.0045
	Cardiac	75.82 ± 0.091	80.88 ± 0.085	81.42 ± 0.079	76.06 ± 0.06
	Fetal	77.54 ± 0.61	78.22 ± 0.64	80.21 ± 0.78	78.25 ± 0.82

**Table 3:** Optimal policies selected by our Med-Aug search algorithm with the reduced BraTS dataset (Policies for the other datasets found by Med-Aug are listed in the Appendix B and Appendix C).

Model	Transform 1	Probability	Magnitude	Transform 2	Probability	Magnitude
U-Net	Sharpness	0.44	5.39	Rotate	0.59	3.09
	Invert	0.99	n/a	ShearX	0.62	3.68
	Elastic Deform	0.85	5.84	Brightness	0.71	0.959
U-Net-Res	Sharpness	0.26	4.87	Solarize	0.65	1.57
	Auto Contrast	0.07	n/a	Bias	0.53	4.24
	Elastic Deform	0.06	5.85	Contrast	0.09	0.427
V-Net	Bias	0.11	1.7	Auto Contrast	n/a	8.32
	TranslateX	0.96	1.9	TranslateX	0.53	0.566
	Elastic Deform	0.001	1.06	Posterize	0.82	1.07
SegResNet	Sharpness	0.83	7.50	Solarize	0.88	3.42
	Auto Contrast	n/a	7.21	Bias	0.99	3.15
	Elastic Deform	0.18	8.09	Contrast	0.13	4.99

**Table 4:** Average *p*-values comparing performance of each model with different sets of DA using BIPOP-CMA-ES and BO-Aug.

Dataset	Accuracy with Basic DA vs. Optimal Policy for BO-Aug (*p*-Value)	Accuracy with Basic DA vs. Optimal Policy for Med-Aug (*p*-Value)
BraTS	1.25 × 10^3^	8.39 × 10^4^
Cardiac	2.00 × 10^3^	1.13 × 10^3^
Fetal	2.04 × 10^3^	2.11 × 10^3^

**Table 5:** BraTS transferability experiments showing the DSC test performance of neural networks trained using optimal policies found from the search with the other neural networks. (IoU version of this table is found in Appendix D).

Model	U-Net	U-Net-Res	V-Net	SegResNet
U-Net Optimal Policy	65.92	65.61	65.65	65.70
U-Net-Res Optimal Policy	65.27	65.92	67.84	67.95
V-Net Optimal Policy	65.23	66.97	68.46	68.29
SegResNet Optimal Policy	65.01	67.64	67.20	68.81

**Table 6:** Evaluation on performance with BraTS and reduced BraTS using our medical DA search. (IoU version of this table is found in Appendix E).

Dataset	Model	Computational Search Time (Hours)	DSC with Optimal Policy) (Mean ± Stdv)
BraTS	U-Net	263.52	66.01 ± 0.0068
	U-Net-Res	285.21	65.67 ± 0.0077
	V-Net	258.65	67.20 ± 0.0041
	SegResNet	292.44	64.22 ± 0.0025
Reduced BraTS	U-Net	75.95	65.92 ± 0.0026
	U-Net-Res	77.54	65.92 ± 0.0034
	V-Net	72.10	68.46 ± 0.0036
	SegResNet	95.83	68.81 ± 0.78

## Data Availability

Not applicable.

## Appendix A

**Table A1:** Optimal policies selected by our Med-Aug search algorithm with the Cardiac dataset.

Model	Transform 1	Probability	Magnitude	Transform 2	Probability	Magnitude
U-Net	Sharpness	0.54	7.69	Solarize	0.90	2.60
	Auto Contrast	n/a	8.03	Bias	0.02	2.76
	Elastic Deform	0.01	4.23	Contrast	0.47	1.46
U-Net-Res	ShearX	0.08	0.16	Motion	0.91	3.97
	TranslateY	0.06	1.88	Solarize	0.09	8.34
	Motion	0.51	6.26	Motion	0.09	8.48
V-Net	Sharpness	0.76	5.28	Solarize	0.95	3.56
	Auto Contrast	n/a	8.29	Bias	0.71	8.29
	Elastic Deform	0.63	5.01	Brightness	0.39	1.14
SegResNet	Sharpness	0.99	4.20	Posterize	0.38	4.64
	Invert	0.99	8.07	ShearX	0.026	2.56
	Elastic Deform	0.15	5.03	Sharpness	0.52	0.19

## Appendix B

**Table A2:** Optimal policies selected by our Med-Aug search algorithm with the Fetal MRI dataset.

Model	Transform 1	Probability	Magnitude	Transform 2	Probability	Magnitude
U-Net	Shear X	0.63	0.33	Motion	09.422	5.21
	Elastic Deform	0.62	6.36	Shear X	0.73	2.26
	Sharpness	0.07	6.18	Contrast	0.06	9.65
U-Net-Res	Ghost	0.21	4.22	Rotate	0.92	7.42
	ShearX	1.22	8.62	Sharpness	0.42	4.16
	ShearX	0.12	5.35	TranslateY	0.56	1.26
V-Net	ShearY	0.61	4.46	Sharpness	0.63	6.12
	Invert	0.12	5.31	ShearY	0.61	6.32
	Sharpness	0.44	6.12	Solarize	0.24	7.12
SegResNet	Sharpness	0.29	6.41	Elastic Deform	0.61	2.36
	Invert	0.76	5.61	Bias	0.001	6.12
	Solarize	0.61	2.57	Auto Contrast	n/a	2.52

## Appendix C

**Table A3:** Testing performance in IoU of four different neural networks using different DA policies on the training set.

Model	Dataset	IoU with no DA (Baseline) (Mean ± Stdv)	IoU with Basic DA (Mean ± Stdv)	IoU with Med-Aug Optimal Policies (Mean ± Stdv)	IoU with BO-Aug Optimal Policies (Mean ± Stdv)
U-Net	BraTS	37.62 ± 0.0001	38.26 ± 0.0005	46.16 ± 0.0013	49.21 ± 0.0044
	Cardiac	70.9 ± 0.0086	71.17 ± 0.011	72.25 ± 0.018	74.96 ± 0.015
	Fetal	72.91 ± 0.35	74.17 ± 0.26	74.92 ± 0.0017	74.23 ± 0.0035
U-Net-Res	BraTS	37.76 ± 0.0006	38.26 ± 0.0005	49.16 ± 0.0017	49.62 ± 0.0035
	Cardiac	50.61 ± 0.043	52.77 ± 0.043	54.34 ± 0.051	63.03 ± 0.0096
	Fetal	70.23 ± 0.49	71.07 ± 0.53	73.03 ± 0.626	71.66 ± 0.51
V-Net	BraTS	42.8 ± 0.0013	43.32 ± 0.0008	52.05 ± 0.0018	50.73 ± 0.0038
	Cardiac	50.61 ± 0.014	52.77 ± 0.018	54.34 ± 0.022	54.12 ± 0.032
	Fetal	66.06 ± 0.28	69.81 ± 0.33	71.90 ± 0.34	69.56 ± 0.29
SegResNet	BraTS	43.17 ± 0.001	43.67 ± 0.0012	53.74 ± 0.0015	47.84 ± 0.0023
	Cardiac	61.06 ± 0.048	67.9 ± 0.044	68.66 ± 0.041	61.37 ± 0.031
	Fetal	63.32 ± 0.44	64.23 ± 0.47	66.96 ± 0.64	64.27 ± 0.69

## Appendix D

**Table A4:** BraTS transferability experiments showing the IoU test performance of neural networks trained using optimal policies found from the search with the other neural networks.

Model	U-Net	U-Net-Res	V-Net	SegResNet
U-Net Optimal Policy	49.16	48.82	48.86	48.92
U-Net-Res Optimal Policy	48.45	49.16	51.33	51.46
V-Net Optimal Policy	48.4	50.34	52.05	51.85
SegResNet Optimal Policy	48.16	51.10	50.60	52.45

## Appendix E

**Table A5:** Evaluation on performance with BraTS and reduced BraTS using our medical DA search.

Dataset	Model	Computational Search Time (Hours)	IoU with Optimal Policy) (Mean ± Stdv)
BraTS	U-Net	263.52	49.26 ± 0.0034
	U-Net-Res	285.21	48.89 ± 0.0039
	V-Net	258.65	50.60 ± 0.0021
	SegResNet	292.44	47.30 ± 0.0013
Reduced BraTS	U-Net	75.95	49.16 ± 0.0013
	U-Net-Res	77.54	49.16 ± 0.0017
	V-Net	72.10	52.05 ± 0.0018
	SegResNet	95.83	52.45 ± 0.6393
